# The Regulation of Apoptosis in Kidney Development: Implications for Nephron Number and Pattern?

**DOI:** 10.3389/fped.2014.00128

**Published:** 2014-11-18

**Authors:** Jacqueline Ho

**Affiliations:** ^1^Department of Pediatrics, Division of Nephrology, University of Pittsburgh School of Medicine, Pittsburgh, PA, USA

**Keywords:** kidney development, apoptosis, nephron number, nephron pattern, cystic kidney disease

## Abstract

Apoptosis is essential to remodel developing structures and eliminate superfluous cells in a controlled manner during normal development, and continues to be an important component of tissue remodeling and regeneration during an organism’s lifespan, or as a response to injury. This mini review will discuss recent studies that have provided insights into the roles of apoptosis in the determination of nephron number and pattern, during normal and abnormal kidney development. The regulation of congenital nephron endowment has implications for risk of chronic kidney disease in later life, whereas abnormalities in nephron pattern are associated with congenital anomalies of the kidney and urinary tract (the leading cause of renal disease in children). Tight regulation of apoptosis is required in normal renal morphogenesis, although many questions remain regarding the regulation of apoptosis by genetic, epigenetic, and environmental factors, in addition to the functional requirement of different components of the apoptotic pathway.

Apoptosis is a process used by multicellular organisms to dispose of unwanted cells in a controlled manner that minimizes damage and disruption to neighboring cells [reviewed in Ref. (1)]. In the context of development, apoptosis is an essential component in the establishment of tissue architecture, and serves to both remodel developing structures to their mature form and ablate superfluous cells [reviewed in Ref. (2)]. Moreover, apoptosis plays a crucial role in tissue remodeling and regeneration that occurs constitutively, or in response to insult or injury, throughout the lifespan of vertebrates. Thus, dysfunctions in apoptosis can manifest as developmental abnormalities, and in adult pathologies such as cancer or degenerative disease.

In the genitourinary tract, the most obvious examples of a requirement for apoptosis during development include the regression of the pronephros and the selective loss of portions of the mesonephros in females during kidney development (3, 4). In addition to these examples, studies have implicated apoptosis more generally in determining nephron number and pattern. Since the mammalian kidney is unable to compensate for nephron loss due to renal injury by the *de novo* generation of nephrons, the number of nephrons generated at birth in any one individual is thought to be an important determinant of adult kidney health (5, 6). In keeping with this concept, reduced nephron number has been associated with hypertension and chronic kidney disease in human beings (7, 8). Moreover, the function of the mammalian kidney is critically dependent on the complex structural arrangement of multiple cell types in the nephron (or nephron pattern) within the kidney itself. This review will discuss recent studies that have provided insights into the roles of apoptosis in the determination of nephron number and pattern during normal and abnormal kidney development.

## The Mechanics of Apoptosis

Cells undergoing apoptosis are characterized by a series of distinct morphological events (cell shrinkage and retraction from neighboring cells, blebbing of the plasma membrane, and cellular fragmentation into apoptotic bodies) that ultimately result in these cells being rapidly engulfed by phagocytes (9). These events are accompanied by subcellular changes, such as nuclear condensation and DNA fragmentation in apoptotic cells. Together, these morphological changes are thought to distinguish specific cells for removal in a controlled fashion, without the activation of inflammatory cells (in contrast to cell death attributed to necrosis). A wide variety of normal and pathological stimuli have been identified that trigger apoptosis, and are thought to do so through two pathways: the intrinsic and the extrinsic pathways.

**Figure 1:**
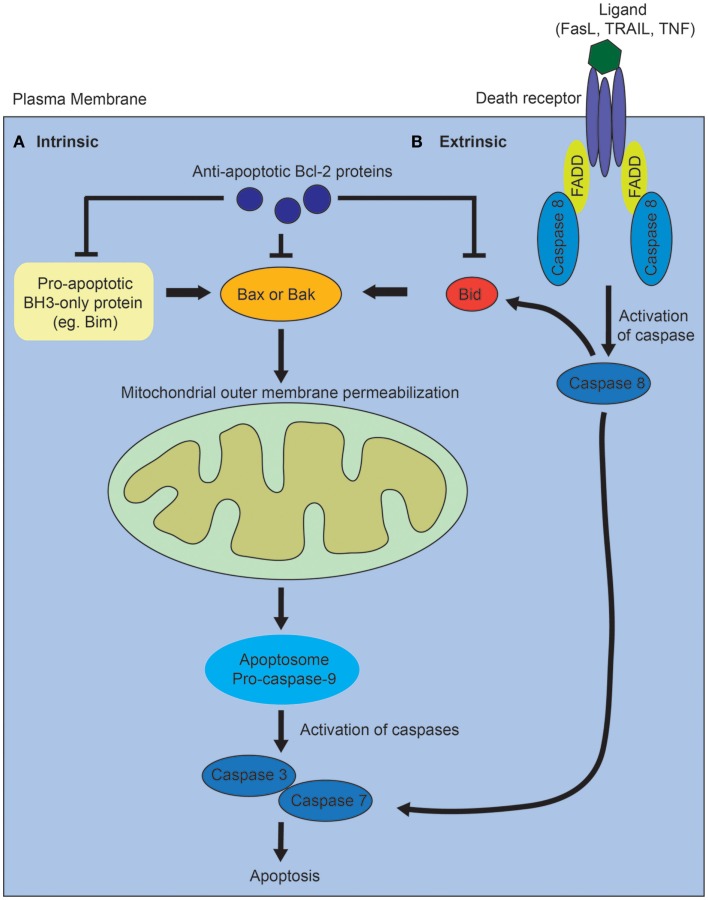
**Diagram of intrinsic and extrinsic pathways of apoptosis**. **(A)** In the intrinsic pathway, the proapoptotic BH3-only family members activate Bax or Bak, leading to mitochrondrial outer membrane permeabilization, which drives formation of the apoptosome, activation of the executioner caspases, 3 and 7, and subsequent apoptosis. The proapoptotic BH3-only proteins are inhibited via interactions with the anti-apoptotic Bcl-2 family of proteins. **(B)** In the extrinsic pathway, ligands such as Fas, tumor necrosis factor (TNF), or tumor necrosis factor-related apoptosis-inducing (TRAIL) ligand bind to death receptors. This results in the recruitment of Fas-associated death domain protein (FADD) and activation of caspase 8. Caspase 8 directly activates caspase 3 and 7. The two pathways interact via caspase 8-mediated cleavage of Bid.

Activation of the intrinsic pathway is regulated via complex interactions between the pro- and anti-apoptotic members of the B-cell lymphoma 2 (Bcl2) family of proteins, and it is thought that it is the balance between these factors that determines whether a cell will undergo apoptosis (Figure 1A) [reviewed in Ref. (10)]. The proapoptotic Bcl2 family proteins are divided into effector proteins (Bax, Bak), which are required for mitochondrial outer membrane permeabilization, and the BH3-only proteins, which either interact with the anti-apoptotic Bcl2 members, or the effector proteins. The anti-apoptotic Bcl2 family members inhibit apoptosis by binding proapoptotic Bcl2 family members and activated Bax or Bak. Mitochondrial outer membrane permeabilization leads to the release of proapoptotic proteins from the mitochondrial intermembrane space, and is the crucial event that drives subsequent activation of caspases via a protein complex termed the apoptosome. The eventual outcome is activation of the executioner caspases, 3 and 7, and subsequent apoptosis.

In contrast, the extrinsic apoptotic pathway is initiated when a death receptor (Fas, tumor necrosis factor receptors) is bound by its ligand (Figure 1B) [reviewed in Ref. (10)]. This results in the subsequent recruitment of adaptor proteins such as Fas-associated death domain protein and procaspase 8. Activated caspase 8 directly cleaves and activates the executioner caspases, 3 and 7. The intrinsic and extrinsic pathways also interact via caspase 8-mediated cleavage of Bid, which leads to mitochondrial outer membrane permeabilization. In vertebrates, most apoptotic stimuli are thought to require mitochondrial outer membrane permeabilization for caspase activation and apoptosis. The relative contribution of the extrinsic versus intrinsic pathways of apoptosis during normal kidney development remains unclear.

## Apoptosis in the Establishment of Nephron Number

**Figure 2:**
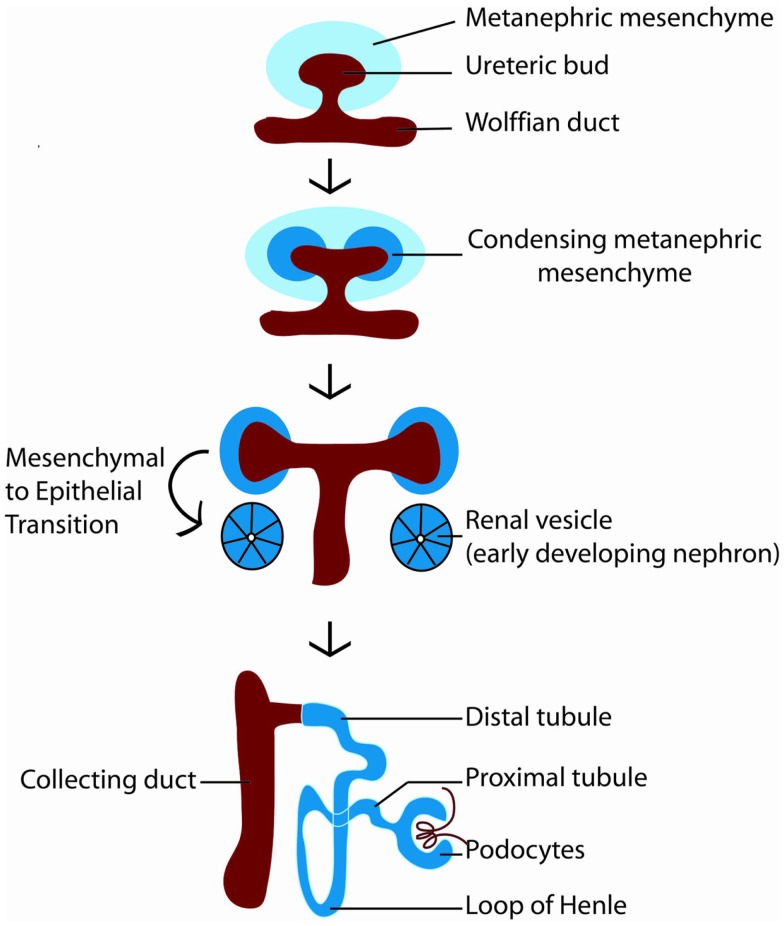
**Diagram of kidney development**. Kidney development begins with the outgrowth of the ureteric bud (red) from the Wolffian duct into the metanephric mesenchyme (light blue). In response to signals from the ureteric bud, the metanephric mesenchyme condenses around the ureteric bud tip, becoming specified as nephron progenitors (dark blue). Nephron progenitors give rise to multiple cell types of the nephron, including podocytes, proximal tubules, loops of Henle, and distal tubules. The ureteric bud branches in response to signals from the metanephric mesenchyme to form the collecting system of the kidney.

How is nephron number determined? The mature mammalian kidney arises from reciprocal interactions between two tissues derived from the intermediate mesoderm: the ureteric bud and the metanephric mesenchyme (11). A series of inductive signals between these two tissues result in the iterative branching of the ureteric bud (which will subsequently become the collecting system of the kidney), and the formation of a “cap” of nephron progenitors (which are fated to become nephrons) around each ureteric bud tip (Figure 2). These nephron progenitors possess the ability to proliferate and self-renew throughout development, to generate an appropriate number of nephrons, and to differentiate into the multiple cell types required to form a functioning nephron (12, 13). The process of new nephron formation continues until around 36 weeks of gestation in human beings, and then terminates, via mechanisms that are largely unknown. Interestingly, the number of nephrons that are created appears to be limited by the number of nephron progenitors generated during kidney development. Thus, ablation of a subset of nephron progenitors results in decreased ureteric bud branching and adult nephron endowment (14). The converse is also true, in which mutations that affect ureteric bud branching result in impaired adult nephron endowment (15–21).

Taken together, these findings suggest that mechanisms that regulate apoptosis of the metanephric mesenchyme (from which nephron progenitors are derived), and of the progenitors themselves, would have a significant impact on congenital nephron endowment. Conceptually, those mechanisms could be considered broadly into two categories: (1) apoptosis resulting from paracrine or autocrine signals to eliminate unwanted cells; or (2) apoptosis as the result of a default intrinsic pathway from which some cells are rescued by survival signals. Classic studies using embryonic kidney explants demonstrated that isolated metanephric mesenchyme undergoes apoptosis in the absence of inducing signals from the ureteric bud (22). Subsequent work to characterize the growth factors that promote survival of the metanephric mesenchyme or nephron progenitors *in vitro* have identified several candidates including transforming growth factor-β2 (TGF-β2), TGFα, leukemia inhibitory factor (LIF), epidermal growth factor (EGF), fibroblast growth factor 2 (FGF2), FGF9, FGF20, and bone morphogenetic protein 7 (BMP7) (23–27). More recently, *in vivo* transgenic mouse studies showed that *Fgf9* and *Fgf20* are critical for maintaining nephron progenitor survival; furthermore, FGF20 mutations in human beings were shown to be associated with severe renal dysplasia (28). The extent to which some or all of these factors act endogenously to promote nephron progenitor survival remains to be determined.

In keeping with the idea that the metanephric mesenchyme is programed to undergo apoptosis is the observation that an inability to respond to inducing and/or survival signals also results in apoptosis. Thus, conditional deletion of both *Fgfr1/Fgfr2* in the metanephric mesenchyme causes marked apoptosis in the metanephric mesenchyme early in kidney development (19). Another example is that of the transcription factor, Wilms tumor-1 (Wt1), which is one of several transcription factors thought to be critical in specification of nephron progenitors from the metanephric mesenchyme. Wt1 null mice have an intrinsic defect in the metanephric mesenchyme that results in abnormal formation of this tissue, which subsequently undergoes rapid apoptosis (29). Finally, impairment of the cell–cell interactions between the metanephric mesenchyme and the ureteric bud can result in subsequent apoptosis of the mesenchyme, as seen in alpha8-integrin null mice (30).

Recent studies have also implicated epigenetic mechanisms (defined as heritable changes in gene activity that are not caused by changes in DNA sequence) in regulating apoptosis in nephron progenitors. microRNAs (miRNAs) are endogenous, small non-coding RNAs that bind to specific mRNA targets to block translation and/or promote mRNA degradation. Conditional targeting of *dicer*, an enzyme required for processing of mature miRNAs, in mouse nephron progenitors, led to premature depletion of nephron progenitors due to excessive apoptosis, likely from upregulation of the proapoptotic protein, Bim (31). In addition to genetic and epigenetic factors, there is also emerging data suggesting that there are environmental influences that impact apoptosis of the metanephric mesenchyme. Thus, disruption of the G protein-coupled bradykinin B2 receptor gene (*Bdkrb2*) in combination with gestational high salt results in increased apoptosis of the metanephric mesenchyme (32). Together, these data suggest that multiple factors (epigenetic, genetic, and environmental) converge to regulate apoptosis in nephron progenitors, with consequent effects on congenital nephron endowment, and thus, the risk of chronic kidney disease in human beings.

## Nephron Pattern and Cell Death

Apart from its role in the determination of nephron number, apoptosis is tightly regulated in kidney development to eliminate unwanted cells as nephrons and the surrounding tissues are patterned to form the complex three-dimensional architecture of the kidney. Experimental surveys of apoptosis in the normal developing kidney have identified a distinct developmental time course for apoptosis in two main areas of the developing kidney: the nephrogenic zone (where new developing nephrons are produced) and the medullary papilla (which gives rise to the calyces, renal pelvis, and renal papilla) (33–35). Interestingly, the cell death rate was highest in the nephrogenic zone of embryonic rat kidneys, concurrent with high rates of cell proliferation, and declined postnatally, suggesting tight regulation of both proliferation and cell survival in this compartment during the process of nephron formation (33). In contrast, the cell death rate in the rat medullary papilla peaked at around postnatal day 6–7, with a subsequent decline (33). In terms of location, most of the apoptotic cells detected in the nephrogenic zone were in the stromal mesenchyme surrounding nephron progenitors and developing nephrons, with fewer apoptotic nephron progenitors (33). In the developing collecting system, apoptosis is infrequently detected in the ureteric bud and is prominent in the medullary papillary region (33). This suggests a potential role for apoptosis in remodeling the first 3–5 generations of the branched ureteric bud/developing collecting duct system. Other suggested roles for medullary apoptosis include elimination of interstitial cells as a mechanism for making room for new blood vessel ingrowth (36).

During nephron formation, nephron progenitors undergo a mesenchymal to epithelial transition to form the renal vesicle, which subsequently differentiates into the comma-shaped body, followed by the S-shaped body, and then the mature nephron (11). Survival signals are equally important in this process, which establishes proximal–distal pattern of the developing nephron. One example is that loss of FGF8 signaling in the metanephric mesenchyme results in progressive loss of the progenitor population, along with a failure to form S-shaped bodies (37). Interestingly, mice that are hypomorphic for FGF8 do develop S-shaped bodies; however, the nephrons that form are truncated due to apoptosis (37).

Evidence that apoptosis is regulated during renal branching morphogenesis is provided by studies in which dysregulated apoptosis is associated with defective collecting duct development. For example, increased branched ureteric bud cell proliferation and subsequent medullary collecting duct cell apoptosis was observed in *Glypican 3^−/−^* (*Gpc3)* mice, which exhibit cystic degeneration of the medullary collecting duct system (38, 39). The defect is thought to be caused by an altered cellular response to growth factors, such as FGFs (39–41). Moreover, apoptosis is a prominent feature of dilated collecting ducts in experimental models of fetal and neonatal urinary tract obstruction (42, 43). Together, these data suggest a relationship between collecting duct apoptosis and two frequent features of renal dysplasia – cystogenesis and urinary tract dilatation.

## The Apoptotic Machinery in the Kidney

Despite the evidence that apoptosis plays a critical role in multiple facets of kidney development, there have been few studies that implicate specific members of the apoptotic pathway in renal morphogenesis. This is thought to be due to functional redundancy among the molecules in the apoptotic pathway, in addition to other levels of compensation for the loss of any one single component. Thus, overall suppression of apoptosis via pharmaceutical means to inhibit caspase-9 or caspase-3 activity leads to decreased ureteric branching and nephron formation in embryonic kidney explants (44, 45). However, mice that lack caspase-3 display normal cell death and normal kidney development (46).

One notable exception to this is the prosurvival protein, *B-cell lymphoma 2* (*bcl2*), which is expressed in the ureteric bud and metanephric mesenchyme (47). *Bcl2*-null mice demonstrate increased apoptosis in the metanephric mesenchyme, resulting in fewer nephrons, mild renal hypoplasia, and cystogenesis (48–50). As noted above, in broad terms, the determination of whether a cell undergoes apoptosis or survives is dependent on the pairing between Bcl-2 family members that promote cell death and those that promote survival. The proapoptotic protein, Bim, binds to many of the Bcl-2 prosurvival proteins, and is thought to release Bax or Bak proteins from their interaction with Bcl-2 to promote apoptosis (51). Interestingly, the loss of a single *Bim* allele in *Bcl-2* null mice is sufficient to rescue the cystic phenotype of *Bcl-2* null mice, suggesting that gene dosage of *Bim* is critical during nephrogenesis (52). While Bim is expressed in the metanephric mesenchyme, *Bim* null mice do not demonstrate an overt renal structural defect [these mice die at several months of age due to an immune complex glomerulonephritis (53)]. Together, these data suggest that the balance of activity between the prosurvival protein, Bcl-2, and the proapoptotic protein, Bim, regulates subsequent apoptosis of the metanephric mesenchyme via the mitochondrial instrinsic apoptotic pathway. An alternative hypothesis for the requirement for Bcl-2 has also been proposed, in which Bcl-2 interacts with paxillin and focal adhesion kinase (FAK) to bypass the need for integrin-mediated survival signals, allowing cells to migrate as needed for nephrogenesis (54, 55). The relative functional contribution of other Bcl-2 family proapoptotic and prosurvival proteins to nephron progenitor survival or nephron patterning remains unknown.

Apart from components of the “apoptotic machinery,” there are also proteins that are known to regulate apoptosis. One example is the transcription factor and tumor suppressor gene, p53, which activates the expression of proapoptotic factors, such as Bax (56). Mice that are null for p53 exhibit defects in terminal nephron differentiation, and on a C57Bl6 background, a spectrum of congenital anomalies of the kidney and urinary tract (57, 58). While these studies have clearly implicated p53 in renal development, the relative role of p53 in the regulation of apoptosis, cell cycle, and/or DNA repair in nephrogenesis remains to be determined.

## Conclusion

Apoptosis is tightly regulated in space and time during kidney development, and dysregulation of apoptosis is clearly associated with changes in nephron number and pattern. However, many questions remain unanswered, including how do genetic, epigenetic, and environmental factors interact to regulate apoptosis? what is the role of other members of the Bcl-2 family of proteins or other components of the apoptotic cascade in kidney development? what are the relative contributions of the intrinsic versus extrinsic pathways in apoptosis? An improved understanding of these processes would contribute to our understanding of congenial anomalies of the kidney and urinary tract, the leading cause of pediatric chronic kidney disease. Moreover, the study of nephron progenitor survival has implications for novel therapies, given recent research efforts to develop methods to reliably propagate and differentiate nephron progenitors in culture, and potentially manipulate progenitors *in vivo*.

## Conflict of Interest Statement

Jacqueline Ho’s laboratory is supported by grants from the NIH, March of Dimes, and Satellite Healthcare.

## References

[1] Taylor RC Cullen SP Martin SJ. Apoptosis: controlled demolition at the cellular level. Nat Rev Mol Cell Biol (2008) 9(3):231–41. 10.1038/nrm2312 18073771

[2] Meier P Finch A Evan G Apoptosis in development. Nature (2000) 407(6805):796–801 10.1038/35037734 11048731

[3] Bouchard M. Transcriptional control of kidney development. Differentiation (2004) 72(7):295–306. 10.1111/j.1432-0436.2004.07207001.x 15554941

[4] Staack A Donjacour AA Brody J Cunha GR Carroll P Mouse urogenital development: a practical approach. Differentiation (2003) 71(7):402–13 10.1046/j.1432-0436.2003.7107004.x 12969333

[5] Potter EL Normal and Abnormal Development of the Kidney. Chicago, IL: Year Book Medical Publishers Inc (1972).

[6] Rodriguez MM Gomez AH Abitbol CL Chandar JJ Duara S Zilleruelo GE. Histomorphometric analysis of postnatal glomerulogenesis in extremely preterm infants. Pediatr Dev Pathol (2004) 7(1):17–25. 10.1007/s10024-003-3029-2 15255031

[7] Keller G Zimmer G Mall G Ritz E Amann K. Nephron number in patients with primary hypertension. N Engl J Med (2003) 348(2):101–8. 10.1056/NEJMoa020549 12519920

[8] Hoy WE Hughson MD Singh GR Douglas-Denton R Bertram JF. Reduced nephron number and glomerulomegaly in Australian aborigines: a group at high risk for renal disease and hypertension. Kidney Int (2006) 70(1):104–10. 10.1038/sj.ki.5000397 16723986

[9] Kerr JF Wyllie AH Currie AR. Apoptosis: a basic biological phenomenon with wide-ranging implications in tissue kinetics. Br J Cancer (1972) 26(4):239–57. 10.1038/bjc.1972.33 4561027 PMC2008650

[10] Tait SW Green DR. Mitochondria and cell death: outer membrane permeabilization and beyond. Nat Rev Mol Cell Biol (2010) 11(9):621–32. 10.1038/nrm2952 20683470

[11] Saxen L Organogenesis of the Kidney. Cambridge: Cambridge University Press (1987).

[12] Boyle S Misfeldt A Chandler KJ Deal KK Southard-Smith EM Mortlock DP Fate mapping using Cited1-CreERT2 mice demonstrates that the cap mesenchyme contains self-renewing progenitor cells and gives rise exclusively to nephronic epithelia. Dev Biol (2008) 313(1):234–45. 10.1016/j.ydbio.2007.10.014 18061157 PMC2699557

[13] Kobayashi A Valerius MT Mugford JW Carroll TJ Self M Oliver G Six2 defines and regulates a multipotent self-renewing nephron progenitor population throughout mammalian kidney development. Cell Stem Cell (2008) 3(2):169–81. 10.1016/j.stem.2008.05.020 18682239 PMC2561900

[14] Cebrian C Asai N D’Agati V Costantini F. The number of fetal nephron progenitor cells limits ureteric branching and adult nephron endowment. Cell Rep (2014) 7(1):127–37. 10.1016/j.celrep.2014.02.033 24656820 PMC4049224

[15] Kispert A Vainio S Shen L Rowitch DH McMahon AP. Proteoglycans are required for maintenance of Wnt-11 expression in the ureter tips. Development (1996) 122:3627–37. 8951078 10.1242/dev.122.11.3627

[16] Majumdar A Vainio S Kispert A McMahon J McMahon AP. Wnt11 and Ret/Gdnf pathways cooperate in regulating ureteric branching during metanephric kidney development. Development (2003) 130(14):3175–85. 10.1242/dev.00520 12783789

[17] Ohuchi H Hori Y Yamasaki M Harada H Sekine K Kato S FGF10 acts as a major ligand for FGF receptor 2 IIIb in mouse multi-organ development. Biochem Biophys Res Commun (2000) 277:643–9. 10.1006/bbrc.2000.3721 11062007

[18] Qiao J Uzzo R Obara-Ishihara T Degenstein L Fuchs E Herzlinger D. FGF-7 modulates ureteric bud growth and nephron number in the developing kidney. Development (1999) 126:547–54. 9876183 10.1242/dev.126.3.547

[19] Poladia DP Kish K Kutay B Hains D Kegg H Zhao H Role of fibroblast growth factor receptors 1 and 2 in the metanephric mesenchyme. Dev Biol (2006) 291(2):325–39. 10.1016/j.ydbio.2005.12.034 16442091

[20] Sims-Lucas S Cusack B Eswarakumar VP Zhang J Wang F Bates CM. Independent roles of Fgfr2 and Frs2alpha in ureteric epithelium. Development (2011) 138(7):1275–80. 10.1242/dev.062158 21350013 PMC3050660

[21] Zhao H Kegg H Grady S Truong HT Robinson ML Baum M Role of fibroblast growth factor receptors 1 and 2 in the ureteric bud. Dev Biol (2004) 276(2):403–15. 10.1016/j.ydbio.2004.09.002 15581874 PMC4131686

[22] Bard JB. Growth and death in the developing mammalian kidney: signals, receptors and conversations. Bioessays (2002) 24(1):72–82. 10.1002/bies.10024 11782952

[23] Barasch J Qiao J McWilliams G Chen D Oliver JA Herzlinger D. Ureteric bud cells secrete multiple factors, including bFGF, which rescue renal progenitors from apoptosis. Am J Physiol (1997) 273:F757–67. 9374839 10.1152/ajprenal.1997.273.5.F757

[24] Dudley AT Godin RE Robertson EJ. Interaction between FGF and BMP signaling pathways regulates development of metanephric mesenchyme. Genes Dev (1999) 13:1601–13. 10.1101/gad.13.12.1601 10385628 PMC316794

[25] Koseki C Herzlinger D Al-Awqati Q. Apoptosis in metanephric development. J Cell Biol (1992) 119(5):1327–33. 10.1083/jcb.119.5.1327 1447305 PMC2289732

[26] Plisov SY Yoshino K Dove LF Higinbotham KG Rubin JS Perantoni AO. TGF beta 2, LIF and FGF2 cooperate to induce nephrogenesis. Development (2001) 128(7):1045–57. 11245570 10.1242/dev.128.7.1045

[27] Brown AC Adams D de Caestecker M Yang X Friesel R Oxburgh L. FGF/EGF signaling regulates the renewal of early nephron progenitors during embryonic development. Development (2011) 138(23):5099–112. 10.1242/dev.065995 22031548 PMC3210493

[28] Barak H Huh SH Chen S Jeanpierre C Martinovic J Parisot M FGF9 and FGF20 maintain the stemness of nephron progenitors in mice and man. Dev Cell (2012) 22(6):1191–207. 10.1016/j.devcel.2012.04.018 22698282 PMC3376351

[29] Kreidberg JA Sariola H Loring JM Maeda M Pelletier J Housman D WT-1 is required for early kidney development. Cell (1993) 74:679–91 10.1016/0092-8674(93)90515-R 8395349

[30] Müller U Wang D Denda S Meneses JJ Pedersen RA Reichardt LF. Integrin α8β1 is critically important for epithelial-mesenchymal interactions during kidney morphogenesis. Cell (1997) 88:603–13. 10.1016/S0092-8674(00)81903-0 9054500 PMC2711892

[31] Ho J Pandey P Schatton T Sims-Lucas S Khalid M Frank MH The Pro-apoptotic protein Bim is a MicroRNA target in kidney progenitors. J Am Soc Nephrol (2011) 22(6):1053–63. 10.1681/ASN.2010080841 21546576 PMC3103725

[32] Fan H Stefkova J El-Dahr SS. Susceptibility to metanephric apoptosis in bradykinin B2 receptor null mice via the p53-Bax pathway. Am J Physiol Renal Physiol (2006) 291(3):F670–82. 10.1152/ajprenal.00037.2006 16571598

[33] Coles HS Burne JF Raff MC. Large-scale normal cell death in the developing rat kidney and its reduction by epidermal growth factor. Development (1993) 118(3):777–84. 8076517 10.1242/dev.118.3.777

[34] Foley JG Bard JB. Apoptosis in the cortex of the developing mouse kidney. J Anat (2002) 201(6):477–84. 10.1046/j.1469-7580.2002.00114.x 12489759 PMC1570997

[35] Carev D Krnic D Saraga M Sapunar D Saraga-Babic M. Role of mitotic, pro-apoptotic and anti-apoptotic factors in human kidney development. Pediatr Nephrol (2006) 21(5):627–36. 10.1007/s00467-006-0057-y 16568307

[36] Loughna S Landels E Woolf AS Growth factor control of developing kidney endothelial cells. Exp Nephrol (1996) 4(2):112–8. 8673440

[37] Grieshammer U Cebrian C Ilagan R Meyers E Herzlinger D Martin GR. FGF8 is required for cell survival at distinct stages of nephrogenesis and for regulation of gene expression in nascent nephrons. Development (2005) 132(17):3847–57. 10.1242/dev.01944 16049112

[38] Cano-Gauci DF Song H Yang H McKerlie C Choo B Shi W Glypican-3-deficient mice exhibit developmental overgrowth and some of the renal abnormalities typical of Simpson-Golabi-Behmel syndrome. J Cell Biol (1999) 146:255–64. 10.1083/jcb.146.999.255 10402475 PMC2199732

[39] Grisaru S Cano-Gauci D Tee J Filmus J Rosenblum ND. Glypican-3 modulates BMP- and FGF-mediated effects during renal branching morphogenesis. Dev Biol (2001) 231:31–46. 10.1006/dbio.2000.0127 11180950

[40] Jackson SM Nakato H Sugiura M Jannuzi A Oakes R Kaluza V Dally, a *Drosophila glypican*, controls cellular responses to the TGF-ß-related morphogen, Dpp. Development (1997) 124:4113–20. 9374407 10.1242/dev.124.20.4113

[41] Tsuda M Kamimura K Nakato H Archer M Staatz W Fox B The cell-surface proteoglycan dally regulates wingless signalling in *Drosophila*. Nature (1999) 400:276–80. 10.1038/22336 10421371

[42] Chevalier RL. Growth factors and apoptosis in neonatal ureteral obstruction. J Am Soc Nephrol (1996) 7:1098–105. 8866400 10.1681/ASN.V781098

[43] Tarantal AF Han VK Cochrum KC Mok A daSilva M Matsell DG. Fetal rhesus monkey model of obstructive renal dysplasia. Kidney Int (2001) 59:446–56. 10.1046/j.1523-1755.2001.059002446.x 11168926

[44] Araki T Hayashi M Nakanishi K Morishima N Saruta T. Caspase-9 takes part in programmed cell death in developing mouse kidney. Nephron Exp Nephrol (2003) 93(3):e117–24. 10.1159/000069552 12660414

[45] Araki T Saruta T Okano H Miura M. Caspase activity is required for nephrogenesis in the developing mouse metanephros. Exp Cell Res (1999) 248(2):423–9. 10.1006/excr.1999.4424 10222134

[46] Kuida K Zheng TS Na S Kuan C Yang D Karasuyama H Decreased apoptosis in the brain and premature lethality in CPP32-deficient mice. Nature (1996) 384(6607):368–72. 10.1038/384368a0 8934524

[47] LeBrun DP Warnke RA Cleary ML. Expression of bcl-2 in fetal tissues suggests a role in morphogenesis. Am J Pathol (1993) 142(3):743–53. 7681256 PMC1886804

[48] Nagata M Nakauchi H Nakayama K Loh D Watanabe T. Apoptosis during an early stage of nephrogenesis induces renal hypoplasia in bcl-2-deficient mice. Am J Pathol (1996) 148(5):1601–11. 8623928 PMC1861554

[49] Sorenson CM Rogers SA Korsmeyer SJ Hammerman MR. Fulminant metanephric apoptosis and abnormal kidney development in bcl-2-deficient mice. Am J Physiol (1995) 268(1 Pt 2):F73–81. 7840250 10.1152/ajprenal.1995.268.1.F73

[50] Kamada S Shimono A Shinto Y Tsujimura T Takahashi T Noda T Bcl-2 deficiency in mice leads to pleiotropic abnormalities: accelerated lymphoid cell death in thymus and spleen, polycystic kidney, hair hypopigmentation, and distorted small intestine. Cancer Res (1995) 55(2):354–9. 7812968

[51] Ewings KE Wiggins CM Cook SJ. Bim and the pro-survival Bcl-2 proteins: opposites attract, ERK repels. Cell Cycle (2007) 6(18):2236–40. 10.4161/cc.6.18.4728 17881896

[52] Bouillet P Cory S Zhang LC Strasser A Adams JM. Degenerative disorders caused by Bcl-2 deficiency prevented by loss of its BH3-only antagonist Bim. Dev Cell (2001) 1(5):645–53. 10.1016/S1534-5807(01)00083-1 11709185

[53] Bouillet P Metcalf D Huang DC Tarlinton DM Kay TW Kontgen F Proapoptotic Bcl-2 relative Bim required for certain apoptotic responses, leukocyte homeostasis, and to preclude autoimmunity. Science (1999) 286(5445):1735–8. 10.1126/science.286.5445.1735 10576740

[54] Sorenson CM. Interaction of bcl-2 with Paxillin through its BH4 domain is important during ureteric bud branching. J Biol Chem (2004) 279(12):11368–74. 10.1074/jbc.M310079200 14699151

[55] Sorenson CM Sheibani N. Focal adhesion kinase, paxillin, and bcl-2: analysis of expression, phosphorylation, and association during morphogenesis. Dev Dyn (1999) 215(4):371–82. 10.1002/(SICI)1097-0177(199908)215:4<371::AID-AJA8>3.0.CO;2-H 10417825

[56] El-Dahr S Hilliard S Aboudehen K Saifudeen Z. The MDM2-p53 pathway: multiple roles in kidney development. Pediatr Nephrol (2014) 29(4):621–7. 10.1007/s00467-013-2629-y 24077661 PMC3969418

[57] Saifudeen Z Dipp S El-Dahr SS. A role for p53 in terminal epithelial cell differentiation. J Clin Invest (2002) 109(8):1021–30. 10.1172/JCI0213972 11956239 PMC150944

[58] Saifudeen Z Dipp S Stefkova J Yao X Lookabaugh S El-Dahr SS. p53 regulates metanephric development. J Am Soc Nephrol (2009) 20(11):2328–37. 10.1681/ASN.2008121224 19729440 PMC2799183

